# Advances in Skin Whitening Agents: Mechanisms, Clinical Applications, and Future Perspectives

**DOI:** 10.1111/jocd.71199

**Published:** 2026-09-17

**Authors:** Yuanyuan Chen, Enhui Wang, Yize Li, Yanfang Yang, Zhaofeng Zhang, Junbo Wang, Yanfei Jiang, Chunyue Zhao

**Affiliations:** ^1^ Beijing Qingyan Boshi Health Management Co., Ltd Beijing China; ^2^ Department of Nutrition and Food Safety, School of Public Health Peking University Beijing China; ^3^ Beijing's Key Laboratory of Food Safety Toxicology Research and Evaluation Beijing China; ^4^ Institute of Medical Technology Peking University Health Science Center Beijing China

**Keywords:** hyperpigmentation, melanogenesis, melasma, post‐inflammatory hyperpigmentation, thiamidol, tranexamic acid, tyrosinase

## Abstract

**Background:**

Skin hyperpigmentation disorders are common cosmetic and clinical concerns caused by excessive melanin production, abnormal pigment distribution, or persistent pigment after inflammation. Their heterogeneous pathogenesis and tendency to recur complicate treatment, particularly in patients with darker phototypes.

**Aims:**

To summarize the molecular basis of melanogenesis and critically link these mechanisms to current and emerging strategies for the management of hyperpigmentation, with particular emphasis on melasma and post‐inflammatory hyperpigmentation.

**Methods:**

Relevant mechanistic, clinical, and regulatory literature was narratively synthesized, focusing on melanosome biology, human tyrosinase catalysis, MITF‐mediated transcriptional control, topical depigmenting agents, procedural interventions, treatment safety, recurrence, and emerging therapeutic technologies.

**Results:**

Melanogenesis is regulated across melanosome biogenesis, tyrosinase‐dependent melanin synthesis, and MITF‐controlled gene expression. Effective management of melasma and post‐inflammatory hyperpigmentation requires rigorous photoprotection and control of disease‐specific triggers, combined with appropriate topical or procedural therapy. Established agents include hydroquinone, retinoids, azelaic acid, niacinamide, arbutin derivatives, kojic acid, and vitamin C, while thiamidol and tranexamic acid represent increasingly used options. Chemical peels, microneedling, pigment‐selective lasers, and combination approaches may improve refractory pigmentation but are limited by recurrence and treatment‐induced dyspigmentation, particularly in darker phototypes. Regulatory distinctions surrounding hydroquinone and emerging approaches, including human‐tyrosinase‐specific screening, targeted or device‐assisted delivery, standardized imaging, and phenotype‐informed treatment selection, are also considered.

**Conclusions:**

Hyperpigmentation is best managed using a mechanism‐based, multimodal, and maintenance‐oriented strategy rather than isolated suppression of melanin synthesis.

## 1 Introduction

Skin color is primarily determined by the amount, type, and distribution of melanin pigments in the epidermis. Melanin serves as a natural photoprotector, shielding skin from ultraviolet (UV) radiation and reducing UV‐induced DNA damage [1]. Excessive or uneven pigment accumulation, however, contributes to disorders including melasma, solar lentigines, post‐inflammatory hyperpigmentation (PIH), and ephelides, all of which may produce substantial cosmetic concern and quality‐of‐life burden [2].

The expanding demand for more even skin tone has intensified research into melanogenesis and depigmenting interventions. Melanin is synthesized within melanosomes in melanocytes and transferred to neighboring keratinocytes. Tyrosinase (TYR), tyrosinase‐related protein‐1 (TYRP1), tyrosinase‐related protein‐2 or dopachrome tautomerase (DCT), MITF, oxidative signaling, and intercellular melanosome transfer therefore provide several potential therapeutic targets [3, 4, 5].

A clinically useful review must go beyond enzyme inhibition alone. Melasma is chronic and relapse‐prone, PIH requires control of the initiating inflammatory process, visible light can aggravate pigment in susceptible patients, and procedures can themselves provoke dyschromia. Accordingly, this review integrates molecular mechanisms with condition‐specific management, newer agents, procedural therapy, safety, regulation, and future delivery strategies.

The aim is to provide a mechanistically grounded and evidence‐oriented resource for researchers, dermatologists, and cosmetic scientists while distinguishing established clinical evidence from promising but still experimental approaches.

## 2 Melanin: Types, Structure, and Biological Functions

### 2.1 Classification of Melanin

Melanin pigments are broadly classified into two major cutaneous types: eumelanin and pheomelanin [6]. A third type, neuromelanin, is found in specific brain regions but has limited relevance to skin pigmentation [7]. Eumelanin is a brown‐black polymer derived from oxidation and polymerization of L‐3,4‐dihydroxyphenylalanine (L‐DOPA) intermediates and is a major contributor to darker skin and hair color [8, 9]. Pheomelanin is a sulfur‐containing reddish‐yellow pigment generated when dopaquinone reacts with cysteine; compared with eumelanin, it provides less efficient photoprotection and can participate in pro‐oxidant chemistry under some conditions [10, 11]. The relative amounts and distribution of eumelanin and pheomelanin contribute to visible pigment phenotype [12].

### 2.2 Structural Properties

Eumelanin is a heterogeneous polymer assembled largely from 5,6‐dihydroxyindole (DHI) and 5,6‐dihydroxyindole‐2‐carboxylic acid (DHICA) units. Its irregular organization contributes to broad optical absorption and radical‐scavenging behavior [13]. Additional physical properties include metal‐ion binding, semiconducting behavior, and resistance to degradation [14].

### 2.3 Biological Functions

Melanin serves several important biological functions in skin. It absorbs energy across UV and visible wavelengths and dissipates much of that energy non‐radiatively [15, 16]. In keratinocytes, melanin can form supranuclear caps that position pigment above the nucleus and contribute to protection from photodamage [17]. Melanin also participates in redox buffering and can interact with reactive oxygen species generated by radiation and environmental stressors [18]. The melanocyte‐melanin unit further intersects with innate immune signaling, cytokine responses, and local oxidative homeostasis [19, 20].

### 2.4 Melanosome Transfer

Visible pigmentation depends not only on melanin synthesis but also on the transfer, distribution, and processing of melanosomes in keratinocytes. Proposed transfer mechanisms include uptake of melanocyte dendrite material, shed vesicle‐mediated transfer, coupled exocytosis and endocytosis of melanocores, and direct membrane interactions [21]. Pigment globules containing multiple melanosomes have been identified as intermediates in one shed‐vesicle pathway [22]. Differences in transfer efficiency and keratinocyte handling can therefore alter apparent skin pigmentation even without a proportional change in melanocyte number.

## 3 Molecular Biology of Melanogenesis

### 3.1 The Melanogenic Cascade

Melanin synthesis begins with L‐tyrosine. Tyrosinase catalyzes the rate‐limiting hydroxylation of L‐tyrosine to L‐DOPA and the oxidation of L‐DOPA to dopaquinone [4, 23, 24]. When cysteine is abundant, dopaquinone undergoes non‐enzymatic addition reactions that generate cysteinyldopa intermediates and favor pheomelanin synthesis [25]. In the eumelanin branch, dopaquinone cyclizes and is converted to dopachrome. Dopachrome can rearrange toward DHI or be converted by DCT or TYRP2 to DHICA, after which oxidative reactions and polymerization produce eumelanin [26].

### 3.2 Tyrosinase and Related Proteins

Tyrosinase is a copper‐dependent enzyme whose catalytic center contains histidine‐coordinated copper ions [27]. Mutations that disrupt tyrosinase folding, trafficking, or copper‐dependent catalysis can cause oculocutaneous albinism [28].

TYRP1 contributes to efficient eumelanogenesis and supports tyrosinase stability and melanosomal trafficking. Although DHICA oxidase activity is established for murine Tyrp1, the precise catalytic contribution of human TYRP1 remains less certain [29, 30]. DCT or TYRP2 converts dopachrome to DHICA and thereby influences the chemical composition of eumelanin [31].

### 3.3 Regulation by MITF

Microphthalmia‐associated transcription factor (MITF) is a central regulator of melanocyte differentiation and melanogenic gene expression [32]. MITF binds regulatory regions of TYR, TYRP1, and DCT and integrates signals from several pathways [33]. In the canonical tanning response, alpha‐melanocyte‐stimulating hormone (alpha‐MSH) activates melanocortin‐1 receptor (MC1R), increases cAMP, activates protein kinase A, and promotes CREB‐dependent MITF transcription [34]. MAPK signaling can alter MITF phosphorylation and stability [35], WNT signaling stabilizes beta‐catenin and cooperates with MITF‐associated transcription [36], and PI3K/AKT/GSK‐3beta signaling provides additional context‐dependent regulation [37].

### 3.4 Environmental Regulation

**FIGURE 1:**
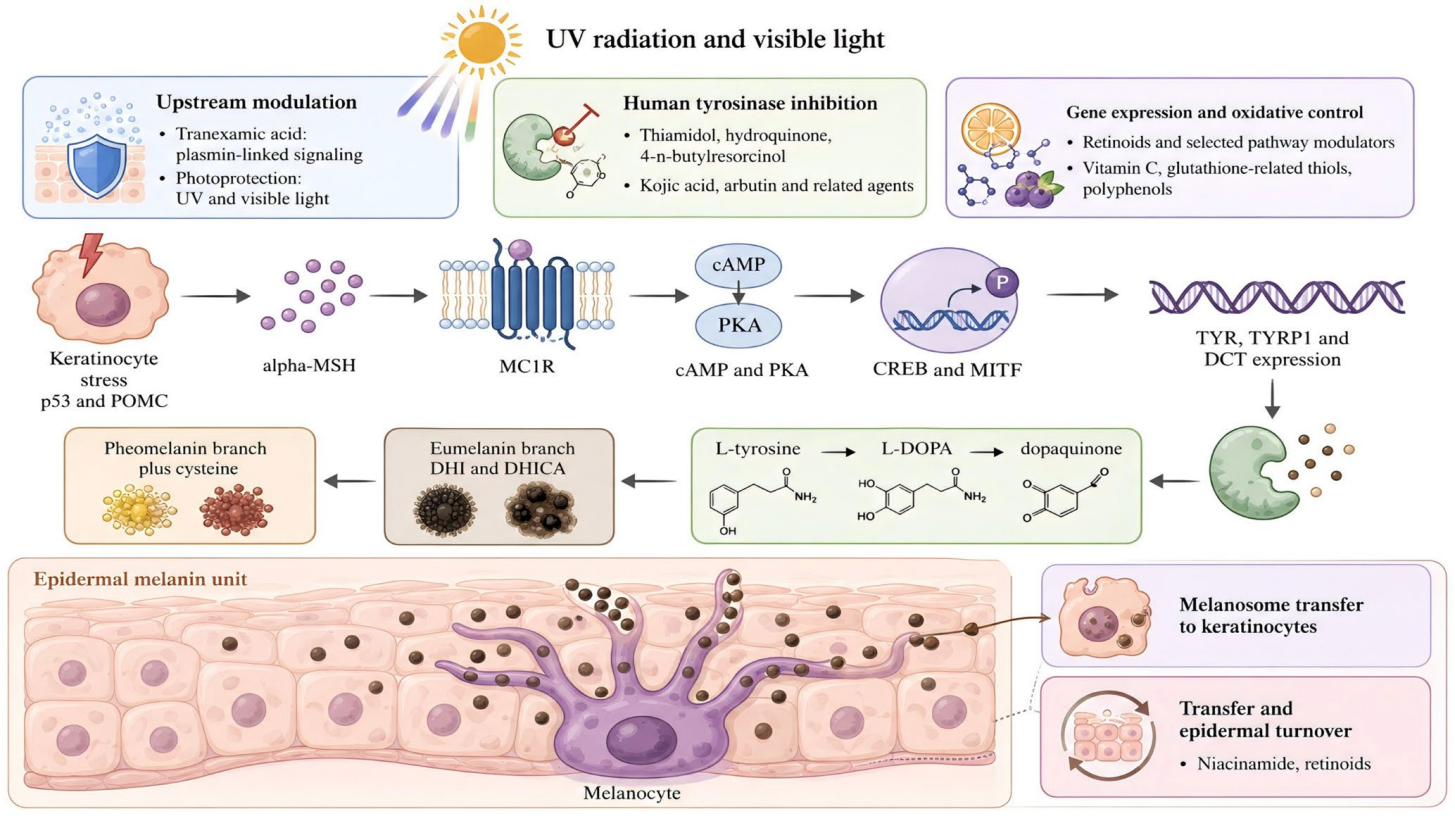
Integrated melanogenesis pathway and major intervention points. The diagram links environmental stimuli, alpha‐MSH and MC1R signaling, MITF‐dependent melanogenic enzymes, melanin synthesis, and melanosome transfer with representative therapeutic targets.

UV radiation stimulates melanogenesis through DNA‐damage responses, oxidative stress, paracrine signaling, and inflammatory mediators [38]. Keratinocyte p53‐dependent induction of POMC and alpha‐MSH provides a major link between UV exposure and melanocyte activation [39]. Sex steroids can modulate pigmentation through non‐classical receptors [40], and cytokines may either promote or inhibit melanogenesis depending on the inflammatory context [41]. Visible light is also clinically relevant, particularly in melasma and darker phototypes, which supports the use of photoprotection strategies extending beyond UV alone [42]. The integrated melanogenic cascade and major therapeutic intervention points are summarized in Figure 1.

## 4 Mechanisms of Skin Whitening and Depigmenting Agents

Depigmenting agents act at multiple levels: direct inhibition of human tyrosinase, modulation of melanogenic transcription, antioxidant or redox effects, interference with melanosome transfer, acceleration of epidermal turnover, and suppression of upstream paracrine signals. Because results from mushroom tyrosinase do not necessarily predict inhibition of human tyrosinase, mechanistic claims should preferentially be supported by human‐enzyme, melanocyte, reconstructed‐skin, or clinical data [43].

### 4.1 Tyrosinase Inhibitors

Hydroquinone has long served as a benchmark depigmenting agent. It interferes with tyrosinase‐dependent melanogenesis and, at higher or prolonged exposure, can produce melanocyte toxicity. Its efficacy is well established, but irritation and exogenous ochronosis limit indiscriminate long‐term use [44, 45, 46]. Arbutin and alpha‐arbutin are hydroquinone‐related glycosides with anti‐melanogenic activity and generally lower irritancy, although formulation quality and control of hydroquinone impurities remain important [47, 48, 49].

Kojic acid, ellagic acid, and several polyphenols can inhibit tyrosinase through copper interaction, redox effects, or additional cell‐signaling mechanisms [50, 51]. Vitamin C does not function simply as a classic competitive tyrosinase inhibitor; rather, it can reduce oxidized intermediates such as dopaquinone and provides antioxidant activity, although instability can limit topical performance [52].

Resorcinol derivatives are increasingly important. 4‐n‐butylresorcinol has demonstrated clinical efficacy in melasma [53]. Thiamidol, or isobutylamido thiazolyl resorcinol, was identified through screening against recombinant human tyrosinase and shows substantially greater potency against human than mushroom tyrosinase [43]. Randomized clinical studies have reported improvement in melasma and broader facial hyperpigmentation, including comparison with hydroquinone and a large vehicle‐controlled study published in 2026 [54, 55, 56].

#### 4.1.1 Human Tyrosinase Specificity and Evidence Translation

A clinically important limitation in depigmenting‐agent discovery is that inhibition of mushroom tyrosinase does not reliably predict inhibition of the human enzyme. Differences in sequence, glycosylation, membrane association, active‐site accessibility, and inhibitor‐binding requirements can change apparent potency across assay systems [43]. For this reason, compounds intended for clinical translation should ideally progress through recombinant human tyrosinase, human melanocyte or reconstructed pigmented‐skin models, and then controlled clinical evaluation rather than being prioritized on mushroom‐enzyme data alone. This human‐specific screening principle is particularly relevant to thiamidol, which was identified through screening against human tyrosinase. A 2024 systematic review of 14 clinical studies found improvement across melasma, PIH, facial hyperpigmentation, and experimentally induced pigmentation, while also emphasizing the need for additional independent and head‐to‐head comparative evidence [57]. The larger 2026 randomized, double‐blind, vehicle‐controlled trial further strengthened the clinical evidence base, but long‐term recurrence, comparative effectiveness, and performance across diverse phototypes remain areas for continued study [56].

### 4.2 Melanosome Transfer Inhibitors

Niacinamide reduces transfer of melanosomes from melanocytes to keratinocytes and has clinical evidence for improving hyperpigmentation [58, 59]. Soybean‐derived serine protease inhibitors can interfere with protease‐activated receptor‐2 signaling and reduce keratinocyte uptake of melanosomes [60]. More recent cellular work also suggests that cytoskeletal and dendrite‐regulating pathways may contribute to the anti‐transfer effects of selected compounds such as glabridin and possibly niacinamide, but translation of these mechanistic findings into clinical superiority remains to be established [61].

### 4.3 Transcriptional and Turnover‐Related Regulators

Retinoids can reduce hyperpigmentation by normalizing keratinocyte differentiation and epidermal turnover and by modulating melanogenic signaling; their use is especially relevant when pigment coexists with acne or photoaging [62, 63]. Glabridin has anti‐melanogenic effects and recent human‐cell work indicates suppression of Wnt/beta‐catenin signaling together with reduced melanocyte dendricity and melanin transfer [61]. Silymarin and catechins such as EGCG influence MAPK, cAMP/CREB/MITF, and oxidative or inflammatory pathways, although the clinical evidence base is less mature than for established agents [64, 65].

### 4.4 Antioxidants and Thiol‐Containing Agents

Oxidative stress amplifies melanogenic signaling, providing a rationale for antioxidant adjuncts. Ascorbic acid can reduce oxidized melanin intermediates [51, 52]. Glutathione and related thiols can react with dopaquinone and may shift melanogenesis toward sulfur‐containing products while also altering cellular redox state; oral, topical, and parenteral approaches have very different evidence and safety profiles and should not be treated as equivalent [66]. N‐acetylcysteine is primarily relevant as a glutathione precursor, and robust comparative clinical evidence for routine depigmenting use remains limited [66].

### 4.5 Tranexamic Acid

Tranexamic acid (TXA) is an antifibrinolytic agent increasingly used off‐label for melasma. Proposed anti‐pigmentary mechanisms include inhibition of plasmin‐related keratinocyte‐melanocyte signaling, reduction of arachidonic‐acid‐derived mediators, effects on vascular or inflammatory components, and modulation of keratinocyte‐derived melanogenic signals. Clinical studies have evaluated oral, topical, intradermal, and microneedling‐assisted delivery. Randomized trials support meaningful improvement in some patients, but recurrence after discontinuation remains common and oral treatment requires careful screening for thromboembolic risk and other contraindications [67, 68, 69].

#### 4.5.1 Route‐Specific Evidence and Patient Selection

The clinical literature on TXA is heterogeneous because oral, topical, intradermal, and microneedling‐assisted regimens are often evaluated with different background therapies and follow‐up intervals. A 2024 systematic review and meta‐analysis of 22 randomized controlled trials involving 1280 patients found significant improvement in melasma severity across TXA routes, with oral regimens showing the largest average reduction in severity scores, but with substantial heterogeneity in treatment combinations and study design [70]. A network meta‐analysis likewise suggested that oral TXA combined with routine topical therapy may produce greater short‐term improvement than several local‐delivery strategies, while also underscoring that route comparisons are limited by inconsistent protocols [71]. These findings support TXA as a clinically relevant adjunct rather than establishing a single universally preferred route.

Patient selection is especially important for oral TXA. Use for melasma remains off‐label, and systemic exposure should be considered only after medical assessment for thromboembolic history, relevant thrombophilia, interacting risk factors, and other contraindications. Local delivery may reduce systemic exposure but does not eliminate uncertainty regarding optimal concentration, treatment interval, or durability. Across routes, recurrence after discontinuation is common, so TXA is best positioned within a broader maintenance plan that includes photoprotection and a tolerable long‐term topical regimen rather than as a stand‐alone finite course [67, 68, 70, 71, 72].

### 4.6 Other Emerging and Multi‐Target Agents

The field is moving from broad screening against mushroom tyrosinase toward compounds validated against human tyrosinase and human skin models [43]. Resveratrol, phenylethyl resorcinol, carotenoid‐related compounds, and other natural or semi‐synthetic agents can combine antioxidant, anti‐inflammatory, and melanogenesis‐modulating actions [73, 74, 75]. However, biochemical activity alone should not be equated with clinical efficacy. Newer candidates should be evaluated for human‐enzyme specificity, skin penetration, irritation potential, stability, and clinically meaningful pigment outcomes. Representative depigmenting agents, their primary mechanisms, typical clinical roles, and key safety considerations are summarized in Table 1.

**TABLE 1:** Representative depigmenting agents and clinically relevant considerations.

Agent	Primary mechanism	Typical clinical role	Key limitations and safety considerations
Hydroquinone	Tyrosinase‐dependent melanogenesis; melanocyte effects	Melasma, PIH and focal hyperpigmentation; strong historical clinical evidence	Irritation, exogenous ochronosis with inappropriate prolonged use; prescription and regulatory status varies by region [45, 46, 76]
Thiamidol	Potent inhibitor of human tyrosinase	Melasma and facial hyperpigmentation; randomized clinical evidence [54, 55, 56]	Generally well tolerated in trials; long‐term comparative and independent evidence remains less extensive than for older agents
4‐n‐Butylresorcinol	Tyrosinase inhibition	Melasma and focal hyperpigmentation [53]	Irritation possible; evidence base smaller than hydroquinone
Azelaic acid	Tyrosinase modulation, anti‐inflammatory effects, normalization of keratinization	Melasma and PIH, especially when acne or inflammation coexists	Stinging and irritation can occur; often useful when hydroquinone is unsuitable
Niacinamide	Reduces melanosome transfer; barrier‐supportive effects	PIH and uneven pigmentation [58, 59]	Usually well tolerated; magnitude of effect is modest compared with stronger depigmenting regimens
Retinoids	Epidermal turnover and melanogenic signaling modulation	PIH, acne‐associated pigment, photoaging; component of triple therapy	Irritation can worsen PIH if introduced too aggressively
Tranexamic acid	Plasmin‐linked signaling, inflammatory and vascular pathways	Melasma, usually as an adjunct [67, 68, 69]	Oral use is off‐label and requires contraindication screening; topical and procedural delivery have variable evidence
Kojic acid and arbutins	Tyrosinase inhibition or related redox effects	Cosmetic and adjunctive depigmenting formulations [47, 51]	Formulation stability, irritancy and human‐tyrosinase potency vary
Vitamin C	Antioxidant and reduction of oxidized melanin intermediates	Adjunctive treatment for uneven pigmentation [52]	Oxidation and formulation instability can limit efficacy

## 5 Clinical Applications and Therapeutic Considerations

### 5.1 Melasma

#### 5.1.1 Clinical Phenotype and Disease Activity

Melasma is a chronic, acquired, usually symmetric facial hypermelanosis with a multifactorial pathogenesis. Beyond increased melanogenesis, affected skin may show changes in the basement membrane, photoaging‐associated signaling, vascularity, and inflammatory or mast‐cell activity. Hormonal influences and repeated UV and visible‐light exposure can sustain disease activity [40, 42, 77]. Because relapse is common, treatment should be framed as long‐term control rather than a one‐time pigment removal strategy.

Recent pathobiologic models support viewing melasma as a disorder of the epidermal and dermal microenvironment rather than isolated melanocyte overactivity. In addition to increased melanogenesis, lesional skin can demonstrate basement‐membrane disruption, solar elastosis, vascular changes, mast‐cell activity, senescent fibroblast signaling, oxidative stress, and barrier abnormalities [78]. These changes help explain why apparently successful pigment reduction may not eliminate the biologic tendency to relapse. Clinical assessment should therefore document distribution, disease duration, phototype, pregnancy or hormonal context, occupational and recreational light exposure, prior irritation, treatment history, and evidence of mixed epidermal and dermal involvement. Dermoscopy or other non‐invasive imaging can be considered when pigment depth or treatment response is uncertain, although such tools complement rather than replace clinical diagnosis [78, 79].

#### 5.1.2 Photoprotection and Visible‐Light Control

Photoprotection is foundational. Broad‐spectrum UV protection should be combined with behavioral light avoidance, and tinted products containing visible‐light‐attenuating pigments can be particularly useful in melasma. In a randomized trial, a UV plus visible‐light protective sunscreen improved outcomes when used with hydroquinone compared with UV‐only protection [42].

Visible‐light protection deserves explicit consideration because high‐energy visible wavelengths can promote persistent pigmentation, particularly in darker phototypes and in melasma‐prone skin. Tinted formulations that contain iron oxides or other visible‐light‐attenuating pigments provide broader spectral protection than non‐tinted ultraviolet‐only formulations. A 2025 review concluded that tinted sunscreens generally outperform non‐tinted comparators for visible‐light‐induced pigmentation and can contribute to melasma relapse prevention, while also noting practical limitations such as inadequate shade diversity and the lack of standardized visible‐light protection metrics [80]. In practice, photoprotection should be framed as a daily therapeutic intervention that combines broad‐spectrum UV protection, visible‐light attenuation when appropriate, shade and behavioral measures, and reapplication according to exposure rather than as a background cosmetic recommendation.

#### 5.1.3 Active Treatment, Escalation, and Maintenance

Topical therapy is selected according to severity, tolerance, prior treatment, pregnancy status, phototype, and access. Hydroquinone‐based regimens and modified triple‐combination therapy remain highly effective options [81, 82, 83, 84]. Thiamidol has randomized evidence in melasma, including vehicle‐controlled studies and a head‐to‐head trial against 4% hydroquinone [54, 55]. Azelaic acid and retinoid‐containing regimens are useful alternatives or adjuncts, particularly when inflammation, acne, or hydroquinone intolerance coexist. TXA can be considered for selected refractory or moderate‐to‐severe cases, but oral treatment is off‐label and requires individualized risk assessment [67, 68].

The 2026 international Delphi consensus reinforces this stepwise approach: broad‐spectrum photoprotection is essential, hydroquinone‐based triple‐combination therapy remains a key supervised standard for appropriate patients, and alternatives or adjuncts include agents such as azelaic acid, kojic acid, and oral TXA [72]. Chemical peels and microneedling can be considered as adjuncts when topical therapy alone is insufficient, whereas lasers are generally better reserved for refractory disease because recurrence and procedure‐related dyspigmentation remain important limitations [72]. Thiamidol is increasingly relevant as a human‐tyrosinase‐targeted option, but its evidence base is newer than that of hydroquinone‐based regimens and should be interpreted accordingly [54, 55, 56, 57].

Maintenance should begin conceptually at treatment initiation rather than after clearance. Once satisfactory improvement is achieved, the regimen can be simplified toward the least irritating combination that preserves control, with continuous photoprotection and periodic reassessment of adherence, recurrence triggers, and tolerability. Escalation should be individualized rather than automatic: failure may reflect inadequate photoprotection, ongoing hormonal or inflammatory drivers, poor adherence, mixed dermal disease, or treatment‐induced irritation rather than insufficient tyrosinase inhibition. This framework is important because repeated cycles of aggressive treatment can worsen barrier dysfunction and increase PIH risk without addressing the factors sustaining disease activity [72, 78, 85].

### 5.2 Post‐Inflammatory Hyperpigmentation

#### 5.2.1 Cause‐Directed Management

PIH follows epidermal or dermal inflammation caused by conditions such as acne, eczema, trauma, irritation, or procedures, and it is often more persistent and clinically prominent in darker skin types [86]. The first treatment principle is to control the underlying inflammatory disorder and prevent new lesions. An aggressive depigmenting regimen that causes dermatitis can perpetuate the very inflammatory signaling that drives PIH.

#### 5.2.2 Topical Therapy and Treatment Sequencing

Daily photoprotection, barrier‐supportive skincare, and gradual introduction of pigment‐directed therapy are central. Depending on the cause and patient characteristics, options include retinoids, azelaic acid, hydroquinone where appropriate, thiamidol, niacinamide, and selected antioxidants. Thiamidol has controlled clinical evidence in PIH [76], while a 2024 Phase IV study found that trifarotene used with an appropriate skincare and UV‐protection routine improved acne‐induced hyperpigmentation while simultaneously treating acne [87].

The evidence base for PIH is less uniform than for melasma because studies include different triggers, phototypes, outcome measures, and treatment durations. A 2024 systematic review of 41 studies and 877 patients found that complete clearance was uncommon across treatment categories, while partial response was more frequent with combination therapy, topicals, and laser or energy‐based approaches; worsening of PIH was also reported after energy‐based treatment in a minority of patients [88]. This supports a realistic clinical endpoint of progressive improvement with prevention of new inflammation rather than promising rapid complete clearance. Treatment intensity should be increased only after the inflammatory driver is adequately controlled and the barrier is tolerating the current regimen.

#### 5.2.3 Special Considerations in Skin of Color

Skin of color requires particular attention because melanogenic responses to inflammation can be more pronounced and persistent, and the cosmetic impact of new dyspigmentation can be substantial. A 2024 systematic review focused on skin of color summarized 48 studies including 1356 individuals, most with Fitzpatrick skin types IV to VI, and found that topical retinoids and lasers were among the most frequently reported interventions. Improvement was common but robust complete clearance was limited, and laser treatment could also exacerbate PIH in some patients [89]. Clinically, this favors slower introduction of irritant actives, strict control of acne or eczema, careful barrier support, conservative procedural parameters, test spots when appropriate, and explicit counseling that inflammation induced by the treatment itself may prolong pigmentation. Trials should also report outcomes across phototypes rather than extrapolating results from lighter‐skinned cohorts [88, 89].

### 5.3 Lentigines, Ephelides, and Other Focal Hyperpigmentation

Solar lentigines generally respond better than melasma to focal topical or procedural treatment because they are more discrete lesions, but lesion diagnosis should be secure before cosmetic therapy. Ephelides are strongly influenced by UV exposure and often recur without photoprotection. In any focal lesion that is atypical in color, border, growth pattern, or symptoms, diagnostic evaluation takes priority over depigmenting treatment. Condition‐specific clinical priorities across common hyperpigmentary disorders are summarized in Table 2.

**TABLE 2:** Condition‐specific clinical priorities in common hyperpigmentary disorders.

Condition	Foundation	Topical or systemic options	Procedural options	Main cautions
Melasma	Broad‐spectrum and visible‐light‐aware photoprotection; avoid hormonal or photic triggers where feasible	Hydroquinone or triple combination, thiamidol, azelaic acid, retinoid combinations; selected TXA	Peels, microneedling, pigment lasers or light devices only in selected patients	High recurrence; dyspigmentation after aggressive procedures; oral TXA contraindications
PIH	Treat the initiating acne, eczema, irritation or injury; maintain barrier and UV protection	Retinoids, azelaic acid, hydroquinone where appropriate, thiamidol, niacinamide	Conservative peels or selected lasers after inflammation is controlled	Irritation can prolong PIH; greater procedure‐related risk in darker phototypes
Solar lentigines	Photoprotection and confirmation of benign diagnosis	Focal depigmenting agents	Cryotherapy, lasers, intense pulsed light or peels depending on lesion and phototype	Post‐procedure PIH and recurrence with sun exposure
Ephelides	Consistent UV protection	Topical lightening agents may provide partial improvement	Selected pigment‐targeted procedures	Often recur with renewed UV exposure

### 5.4 Combination Therapy and Maintenance

Combination treatment often outperforms monotherapy because hyperpigmentation is maintained by several processes at once. The original Kligman formula combined hydroquinone, tretinoin, and a corticosteroid [82], whereas modern modified triple‐combination formulations use different concentrations and corticosteroids to improve tolerability [83]. Hydroquinone plus glycolic acid can enhance response but also increase irritation potential [84]. For chronic melasma, maintenance photoprotection and a tolerable long‐term topical regimen are more defensible than abrupt cessation of all therapy because recurrence reflects persistent disease biology rather than a simple withdrawal phenomenon [85].

### 5.5 Procedural Treatments

#### 5.5.1 Chemical Peels

Chemical peels can accelerate epidermal turnover and are commonly used as adjuncts for epidermal or mixed pigment. Glycolic acid, lactic acid, salicylic acid, Jessner solution, and other superficial peels have been studied. A 2025 randomized trial reported greater MASI reduction with 50% glycolic acid than with 80% lactic acid, but both approaches require attention to irritation and PIH risk [90]. Superficial rather than aggressive peeling is generally favored in patients prone to dyspigmentation.

The practical role of peeling is best understood as controlled epidermal turnover that can complement, but not replace, photoprotection and topical suppression of melanogenesis. Superficial protocols are generally preferred for pigment‐prone skin because deeper or highly inflammatory injury increases the probability of prolonged erythema and PIH. Choice of agent should account for phototype, barrier status, active dermatitis or acne, prior PIH after procedures, and the clinician's ability to standardize contact time and post‐procedure care. Direct comparisons between peel types remain limited, so apparent superiority in a single trial should not be generalized across concentrations or patient populations [72, 90].

#### 5.5.2 Microneedling and Device‐Assisted Delivery

Microneedling is used both for controlled remodeling and as a device‐assisted drug‐delivery technique. Trials combining microneedling with topical TXA or with modified Kligman regimens demonstrate that the approach can be useful, but outcomes are heterogeneous and PIH remains a clinically important complication. In a 2024 randomized study, post‐inflammatory hyperpigmentation occurred in a meaningful subset and was associated with darker phototypes and warmer seasons [69].

A 2025 meta‐analysis of randomized controlled trials supports microneedling primarily as an assisted or adjunctive therapy for melasma rather than a replacement for established topical treatment [91]. Proposed advantages include controlled remodeling and enhanced transcutaneous delivery, but studies vary substantially in needle depth, treatment interval, co‐administered agents, endpoint definitions, and phototype distribution. These sources of heterogeneity matter clinically because more aggressive needling does not necessarily translate into better pigment outcomes and may increase inflammatory risk. When microneedling is used to enhance delivery of TXA or other agents, both the device procedure and the delivered compound contribute to efficacy and adverse events, which should be considered when interpreting combination studies [69, 71, 91].

#### 5.5.3 Lasers and Light‐Based Devices

Lasers and light‐based devices can improve melasma or lentigines in selected patients but must be used conservatively. Low‐fluence Q‐switched Nd:YAG, picosecond alexandrite systems, fractional devices, and intense pulsed light have all been studied. Recent randomized studies show improvement with picosecond approaches, including combination with azelaic acid, but longer follow‐up confirms that recurrence and treatment‐related pigmentary change remain major limitations [92, 93]. Procedural therapy should therefore complement, not replace, photoprotection and maintenance topical care.

**FIGURE 2:**
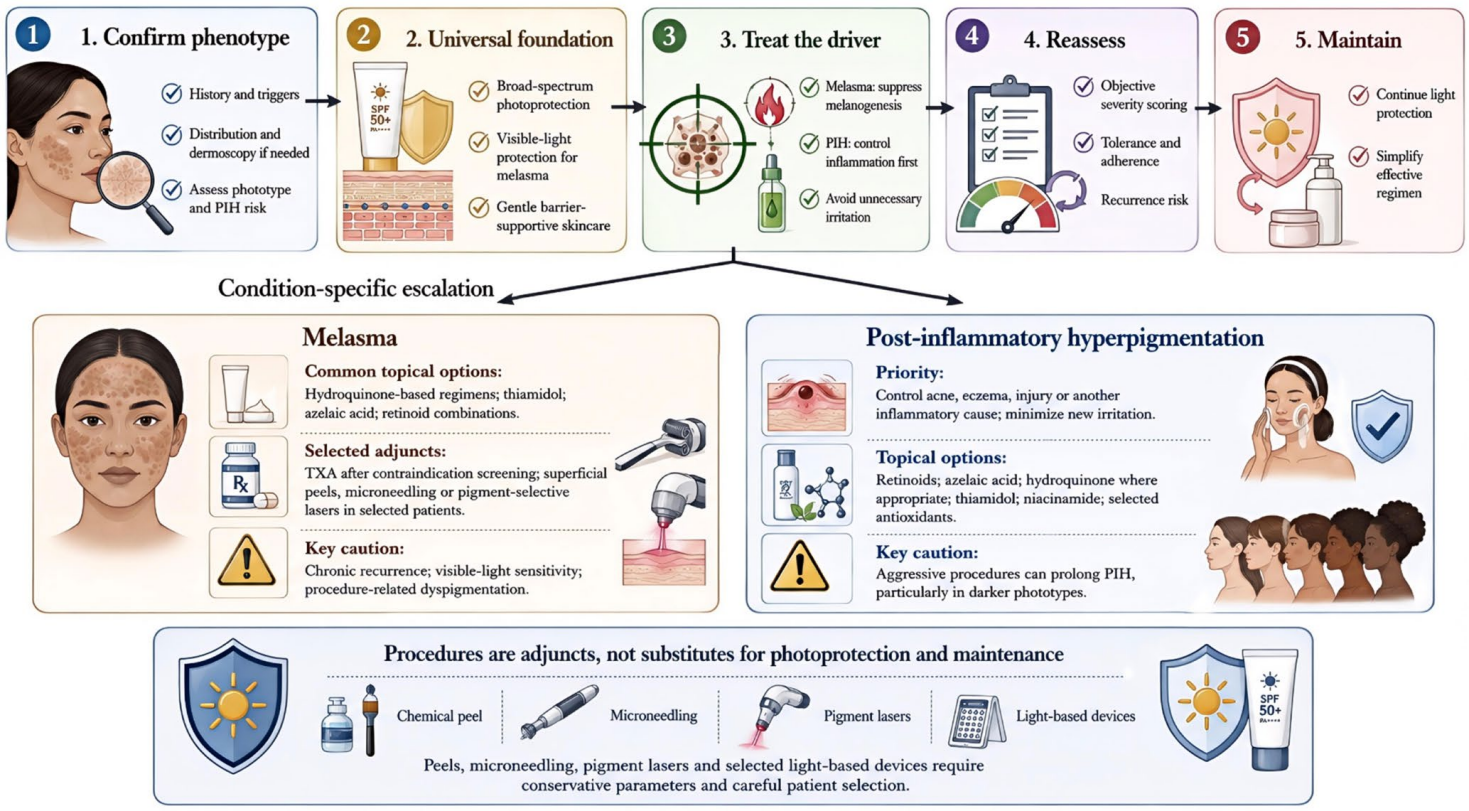
Evidence‐informed clinical framework for common hyperpigmentation. The framework emphasizes phenotype confirmation, photoprotection, treatment of the underlying driver, condition‐specific therapy, cautious procedural escalation, reassessment, and maintenance.

Recent evidence reinforces the need for restrained positioning of laser therapy. A 2026 systematic review and meta‐analysis of randomized trials evaluating 755‐nm picosecond alexandrite laser found that triple‐combination cream produced greater MASI improvement than the laser in the pooled comparison, and the authors highlighted the limited number of trials and need for better long‐term data [94]. Thus, the appeal of rapid pigment targeting should not obscure the chronic biology of melasma. Device choice, wavelength, fluence, pulse structure, interval, cooling, phototype, and baseline inflammatory activity all influence risk. For melasma and PIH, laser or light treatment is most defensible as a selected adjunct for refractory disease under conservative parameters and continued maintenance, not as a universal first‐line strategy [72, 88, 89, 94]. Table 3 summarizes major procedural approaches, and Figure 2 presents the integrated management framework.

**TABLE 3:** Evidence‐informed procedural approaches for hyperpigmentation.

Procedure	Mechanistic rationale	Best clinical role	Evidence position	Key limitations
Superficial chemical peels	Controlled epidermal exfoliation and accelerated turnover	Adjunct for epidermal or mixed melasma and selected PIH after inflammation is controlled	Adjunctive; heterogeneous comparative evidence	Irritation, prolonged erythema and PIH; outcomes depend on agent, concentration and phototype
Microneedling	Controlled dermal injury and enhanced transcutaneous delivery	Adjunctive melasma treatment and device‐assisted delivery of selected topical agents	Growing adjunctive evidence from randomized trials and meta‐analysis	Protocol heterogeneity; pain, erythema and procedure‐induced PIH; contribution of co‐administered drug may be difficult to separate
Low‐fluence Q‐switched Nd:YAG laser	Selective pigment targeting with reduced fluence	Selected refractory melasma in experienced hands	Mixed evidence; generally adjunctive rather than first‐line	Recurrence, mottled hypopigmentation and PIH with repeated or aggressive treatment
Picosecond lasers	Photoacoustic pigment disruption with short pulse duration	Selected refractory melasma or focal pigment, often in combination regimens	Emerging to moderate; no consistent superiority over established topical therapy	Cost, parameter heterogeneity, recurrence and procedure‐related dyspigmentation
Fractional lasers	Fractional remodeling and potential enhancement of topical delivery	Selected mixed or dermal disease and combination protocols	Adjunctive; evidence varies by device and protocol	Inflammation, prolonged erythema, PIH and downtime
Intense pulsed light and other light‐based devices	Broad‐spectrum photothermal targeting of pigment and vascular components	Selected superficial lesions and carefully chosen melasma patients	Selected‐use evidence; highly operator and phototype dependent	PIH, recurrence and risk of worsening melasma if parameters are poorly matched

### 5.6 Safety and Regulatory Considerations

Irritant dermatitis is a common adverse effect across many active depigmenting regimens and can paradoxically worsen PIH. Exogenous ochronosis is a rare but serious complication associated with inappropriate or prolonged hydroquinone exposure [45, 46]. The risk–benefit balance is therefore influenced by concentration, duration, formulation, skin phototype, adherence, and concurrent irritation.

The regulatory status of hydroquinone requires correction from older descriptions. In the United States, there are no legally marketed over‐the‐counter hydroquinone skin‐lightening drugs; after implementation of the CARES Act framework, nonprescription hydroquinone skin‐lightening products without approved applications are considered unapproved drugs. The FDA states that no hydroquinone‐containing skin‐lightening products are approved for over‐the‐counter sale; its 2022 enforcement communication identified Tri‐Luma as an FDA‐approved prescription product containing hydroquinone for short‐term treatment of moderate‐to‐severe facial melasma [95]. In the European Union, hydroquinone is prohibited in cosmetic products for skin lightening, with a narrow professional‐use exception in artificial nail systems under the cosmetics regulation [96]. Regulations in other jurisdictions vary and should be checked using current local sources.

Oral TXA presents a different safety issue because systemic antifibrinolytic exposure can be inappropriate in patients with thromboembolic disease, relevant thrombophilia, or other risk factors. Clinical use for melasma is off‐label and should be based on medical evaluation rather than cosmetic self‐treatment [67, 68].

Because this is a narrative review rather than a formal systematic review, the evidence categories below are intentionally qualitative. They are used to distinguish interventions supported by longstanding comparative clinical experience from newer agents with growing randomized evidence, adjunctive procedures with heterogeneous protocols, and approaches that remain primarily investigational. This distinction is important to avoid presenting biochemical potency, small uncontrolled series, and randomized clinical evidence as equivalent forms of support. The evidence‐informed clinical positioning of major interventions is summarized in Table 4.

**TABLE 4:** Evidence‐informed clinical positioning of major interventions.

Intervention	Primary target or rationale	Best‐supported clinical role	Evidence position	Practical interpretation
Hydroquinone‐based triple combination	Tyrosinase suppression plus retinoid turnover and anti‐inflammatory support	Melasma under medical supervision	Established reference therapy	Highly effective for appropriate patients; irritation and ochronosis risk require controlled duration and monitoring
Azelaic acid	Melanogenesis modulation plus anti‐inflammatory and keratinization effects	Melasma and PIH, particularly with acne or hydroquinone intolerance	Established to moderate clinical evidence	Useful alternative or adjunct with favorable multi‐target rationale; stinging and irritation may limit adherence
Thiamidol	Potent human tyrosinase inhibition	Melasma, PIH and facial hyperpigmentation	Growing randomized clinical evidence	Promising human‐specific inhibitor with expanding trial support; independent long‐term and head‐to‐head evidence remains less extensive than for older standards
Tranexamic acid	Plasmin‐linked signaling with vascular and inflammatory effects	Selected melasma as adjunctive topical, local or oral therapy	Growing clinical evidence with substantial protocol heterogeneity	Oral use is off‐label and requires contraindication screening; recurrence and optimal route remain important questions
Retinoids	Epidermal turnover and melanogenic signaling modulation	PIH, acne‐associated pigment and combination melasma regimens	Established adjunctive evidence	Particularly useful when acne or photoaging coexists; irritation can worsen PIH if escalation is too rapid
Niacinamide	Reduced melanosome transfer and barrier‐supportive effects	PIH and uneven pigmentation	Supportive clinical evidence	Generally well tolerated and useful in combination, but pigment reduction is usually more modest than with stronger depigmenting regimens
Chemical peels	Controlled epidermal exfoliation	Selected epidermal pigment as adjunctive therapy	Adjunctive and heterogeneous	Best used conservatively in pigment‐prone skin; treatment‐induced inflammation can worsen PIH
Microneedling	Remodeling and assisted topical delivery	Adjunctive melasma and combination delivery protocols	Growing adjunctive evidence	Protocol‐dependent; efficacy cannot always be separated from the delivered drug
Lasers and light‐based devices	Selective photothermal or photoacoustic pigment targeting	Selected refractory melasma, lentigines and carefully chosen PIH	Selected‐use evidence with high heterogeneity	Potentially useful but not first‐line for chronic melasma; recurrence and dyspigmentation require conservative patient selection
Nanocarriers and advanced delivery systems	Improved stability, localization and cutaneous delivery	Formulation research and selected translational applications	Emerging; largely formulation or preclinical evidence	Promising for dose reduction and stability, but routine clinical superiority has not been established

## 6 Future Directions and Emerging Research

### 6.1 Human‐Specific Target Discovery

A major methodological shift is the move toward human‐relevant target systems. Compounds that inhibit mushroom tyrosinase can perform very differently against human tyrosinase, so future screening should prioritize recombinant human enzymes, primary or well‐validated human melanocyte models, reconstructed pigmented skin, and clinical confirmation [43]. Structure‐guided optimization of human‐tyrosinase inhibitors may improve potency while reducing non‐specific melanocyte toxicity.

### 6.2 Advanced Delivery Systems

Drug delivery is another active frontier. Liposomes, nanoemulsions, nanostructured lipid carriers, lipid nanoparticles, and device‐assisted delivery aim to improve stability and local epidermal exposure while limiting irritation. Phenylethyl resorcinol has been studied in nanostructured lipid carriers [74], and a 2026 preclinical study reported co‐delivery of glabridin and uralsaponin X using lipid nanoparticles with inhibition of melanogenesis and melanin transfer [97]. These approaches are promising but require rigorous safety, stability, penetration, and comparative clinical testing before routine adoption.

Recent reviews of depigmenting‐active nanosystems and broader dermatologic nanocarriers describe liposomes, nanoemulsions, polymeric systems, solid lipid nanoparticles, and related platforms as strategies to protect unstable actives, control release, and increase local skin exposure [98, 99]. For hyperpigmentation, the most persuasive translational question is not whether a nanocarrier can increase laboratory penetration, but whether it can improve clinically meaningful pigment outcomes while reducing irritation at an equivalent active dose. Future studies should therefore report formulation stability, cutaneous localization, systemic exposure where relevant, comparative tolerability, manufacturing reproducibility, and head‐to‐head clinical outcomes rather than relying on physicochemical characterization alone.

### 6.3 Objective Imaging and Digital Phenotyping

Personalized management is more likely to advance through clinically measurable phenotypes than through near‐term cosmetic gene editing. Useful variables include Fitzpatrick phototype, epidermal versus mixed pigment pattern, inflammatory activity, vascular features, visible‐light sensitivity, prior PIH after procedures, hormonal triggers, and previous treatment response. Standardized photography, colorimetry, dermoscopy, reflectance methods, and validated severity scores can support more reproducible treatment selection and follow‐up, and recent imaging studies support increasingly quantitative assessment of facial pigmentation [100]. Artificial‐intelligence‐assisted melasma assessment has also entered early clinical‐research validation, but external validation across diverse skin tones and settings remains essential before such models can guide treatment decisions [101].

Non‐invasive imaging may help move clinical trials beyond subjective visual grading. Reflectance confocal microscopy can localize pigment depth and has been used to monitor structural and pigmentary changes before and after medical or laser treatment [79]. A 2025 study combined optical coherence tomography with deep learning for melasma diagnosis and follow‐up, illustrating the potential for quantitative imaging to capture tissue‐level changes that are not fully represented by surface photographs [102]. These technologies remain research and specialty tools rather than routine standards, and their future value will depend on external validation, reproducibility across devices, representative training data, and demonstration that imaging‐guided decisions improve patient outcomes rather than simply increasing measurement complexity.

### 6.4 Genome‐Wide Discovery and Personalized Management

Genome‐wide perturbation tools and CRISPR‐based screens are valuable for discovering previously unrecognized regulators of melanogenesis; a genome‐wide human pigmentation screen identified numerous regulators spanning melanosome biogenesis, trafficking, and gene control [103]. Direct gene editing for cosmetic depigmentation remains experimental and raises substantial safety and ethical concerns. Near‐term translation is more likely to come from target discovery, safer small molecules, human‐relevant screening, improved formulations, and rational multimodal regimens.

A realistic near‐term model of personalization is therefore phenotype‐directed rather than genotype‐directed therapy. Patients can be stratified by phototype, epidermal versus mixed pigment distribution, inflammatory activity, visible‐light sensitivity, vascular or photoaging features, prior procedure‐induced PIH, hormonal context, recurrence pattern, and previous treatment response. This information can guide the order and intensity of interventions: for example, prioritizing inflammation control in acne‐associated PIH, visible‐light‐aware photoprotection in melasma, or avoiding early energy‐based escalation in a patient with a strong history of procedure‐induced dyspigmentation. Such stratification should be tested prospectively rather than assumed to improve outcomes.

### 6.5 Durability, Comparative Effectiveness, and Inclusive Trial Design

Finally, future clinical trials should place greater emphasis on durability and recurrence, not only short‐term pigment reduction. Longer follow‐up, standardized reporting of PIH and hypopigmentation, inclusion of darker phototypes, transparent reporting of industry involvement, and head‐to‐head comparisons with established therapies will be important for distinguishing genuinely useful innovations from mechanistically attractive but clinically marginal interventions.

Comparative‐effectiveness research is especially important as the number of marketed depigmenting ingredients and devices increases. Trials should include an established active comparator where ethically and practically appropriate, predefine clinically meaningful change, record adherence and concomitant photoprotection, and distinguish treatment of melasma from treatment of PIH or focal lentigines. Industry‐sponsored studies can contribute valuable data but should report sponsor roles, protocol registration, and adverse events transparently. Greater enrollment of Fitzpatrick skin types IV to VI and reporting by phototype would also improve generalizability for the populations that often experience the greatest burden of PIH and procedure‐related dyschromia [57, 72, 88, 89].

## 7 Conclusions

Skin depigmentation science spans melanosome biology, human tyrosinase catalysis, MITF‐dependent transcription, oxidative and inflammatory signaling, melanosome transfer, and epidermal turnover. Hydroquinone remains an important benchmark treatment, but newer agents such as thiamidol and the expanding evidence for tranexamic acid have broadened the therapeutic landscape. Clinical management is strongest when it is diagnosis‐specific: melasma requires long‐term photoprotection and maintenance because relapse is common, while PIH requires control of the initiating inflammation and avoidance of treatment‐induced irritation. Chemical peels, microneedling, and lasers can add benefit in selected patients but also create pigmentary risk. Future progress will depend on human‐relevant screening, safer multi‐target agents, improved delivery systems, objective outcome measurement, and phenotype‐informed treatment selection. These developments should support more effective and safer management of hyperpigmentation without overstating preclinical findings as established clinical benefit.

## Funding

The authors have nothing to report.

## Conflicts of Interest

The authors declare no conflicts of interest.

## Data Availability

Data sharing not applicable to this article as no datasets were generated or analysed during the current study.
